# Sleep disturbance is associated with neck pain: a 3-year longitudinal study after the Great East Japan Earthquake

**DOI:** 10.1186/s12891-022-05410-w

**Published:** 2022-05-16

**Authors:** Yutaka Yabe, Yoshihiro Hagiwara, Takuya Sekiguchi, Yumi Sugawara, Masahiro Tsuchiya, Shinichirou Yoshida, Ichiro Tsuji

**Affiliations:** 1 Department of Orthopaedic Surgery, Tohoku University School of Medicine, 1-1 Seiryo-machi, Aoba-ku, Sendai, Miyagi 980-8574 Japan; 2 Division of Epidemiology, Department of Health Informatics and Public Health, Tohoku University Graduate School of Public Health, 2-1 Seiryo-machi, Aoba-ku, Sendai, Miyagi 980-8575 Japan; 3 Department of Nursing, Faculty of Health Science, Tohoku Fukushi University, 1-8-1 Kunimi, Aoba-ku, Sendai, Miyagi 981-8522 Japan

**Keywords:** Neck pain, Sleep disturbance, Natural disaster, The Great East Japan Earthquake, Survey, Physical condition, Mental health, Psychological distress, Social network, Economic status

## Abstract

**Background:**

Neck pain is a common health problem in the general population as well as in people after natural disasters. Sleep disturbances are gaining attention as risk factors for musculoskeletal pain; however, the association between sleep disturbance and neck pain has not been clarified. The present study aimed to clarify the association between sleep disturbance and neck pain, especially focusing on the effect of the duration of sleep disturbance, after the Great East Japan Earthquake.

**Methods:**

This study used 3-year longitudinal data obtained from individuals (*n* = 2,059) living in disaster-affected areas after the Great East Japan Earthquake. Sleep disturbance and neck pain were investigated at 4, 5, 6, and 7 years after the disaster. Multivariate logistic regression analyses were used for the assessment.

**Results:**

Sleep disturbance was significantly associated with neck pain, and the association was stronger as the duration of sleep disturbance increased (adjusted odds ratios [95% confidence intervals]: 1.84 [1.23–2.75] for “ < 1 year”; 2.41 [1.53–3.81] for “ ≥ 1 year and < 2 years”; 2.80 [2.09–3.76] for “ ≥ 2 years”). Furthermore, preceding sleep disturbance was significantly associated with the onset of neck pain, and the association was stronger as the duration of sleep disturbance increased (adjusted odds ratios [95% confidence intervals]: 1.86 [1.08–3.20] for “ < 1 year”; 2.39 [1.22–4.70] for “ ≥ 1 year and < 2 years”; 3.00 [1.94–4.65] for “ ≥ 2 years”).

**Conclusions:**

Sleep disturbance is associated with neck pain, and long-lasting sleep disturbance strengthens the association. Clinicians should consider this association to effectively treat patients with neck pain, especially those affected by natural disasters.

## Background

Neck pain is a common health problem worldwide [1]. Generally, other terms are also used in similar symptoms with neck pain, such as neck/shoulder pain in European countries and katakori in Japan [2, 3]. Neck pain is often a cause of disability and identifying the risk factors is important for the treatment of neck pain [4]. Some factors such as age, sex, working status, and psychosocial conditions are reported to be related to neck pain [1, 5–9]. In recent years, sleep disturbances are gaining interest as risk factors for musculoskeletal pain [10–12], and some authors have reported the association between sleep disturbance and neck pain [13–18]. Some cross-sectional studies have shown a high rate of sleep disturbance among people with neck pain [13, 17]. Moreover, some authors have shown that sleep disturbance is a prognostic factor of neck pain in longitudinal studies [15, 16]. Although there have been some reports on this topic, its number is small and the association of sleep disturbance with neck pain is not clarified.

Musculoskeletal pain and sleep disturbance are common problems after natural disasters [19]. The Great East Japan Earthquake (GEJE) attacked the northeast coastal areas of Japan on 11 March 2011, and caused severe damage [20]. Although reports of neck pain after natural disasters are rare, the prevalence of neck pain was reported to be high after the GEJE [21], and deteriorated subjective economic conditions were associated with neck pain onset [22]. Furthermore, stressful conditions after the disaster increase sleep disturbance [23], which may also be associated with neck pain; however, the association between sleep disturbance and neck pain after natural disasters has not been reported. Clarifying the association between sleep disturbance and neck pain is important to develop strategies for the treatment of neck pain among people after natural disasters as well as in general population. The purpose of the present study was to elucidate the association between sleep disturbance and neck pain using 3-year cohort data of people after the GEJE, especially focusing on the effect of the duration of sleep disturbance.

## Methods

### Participants

**Fig. 1:**
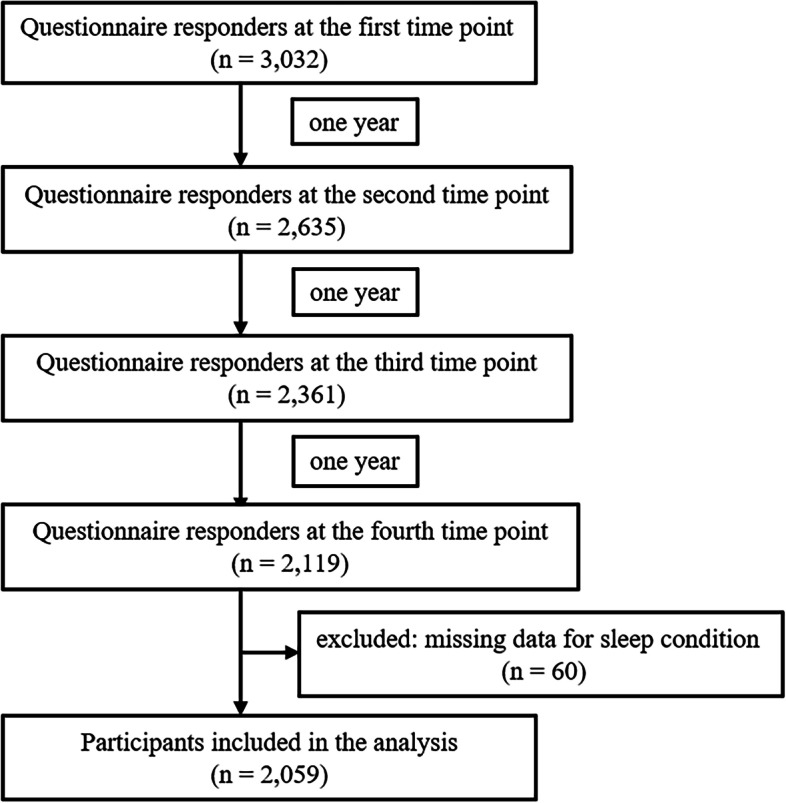
Flow chart of the study

A comprehensive panel study has been conducted with people living in the disaster-affected areas after the GEJE, such as Ogatsu, Oshika, and Ajishima areas in Ishinomaki city and Wakabayashi Ward in Sendai city in Japan. The first survey was conducted 3 months after the GEJE and repeated annually. This cohort aims to assess and support the physical and mental health conditions of people living in these areas since the health system was destroyed by the disaster. The initial population included in the survey were residents registered in the basic residential registry of Ogatsu, Oshika, and Ajishima areas and people living in prefabricated housing in Wakabayashi Ward. The present study used the data at 4, 5, 6, and 7 years after the GEJE (defined as the first, second, third, and fourth time points, respectively) to assess the association between sleep disturbance and neck pain. For each time-point survey, the people who had participated in the survey conducted the previous year were called up (age ≥ 18 years). At the first point, 4,324 people were recruited, and 3,032 responded (70.1%). Among these 3,032 individuals, 2,635 participated in the second-point survey (86.9%). Of the 2,635 people, 2,361 responded to the third-point survey (89.6%). Among these 2,361 individuals, 2,119 participated in the fourth-point survey (89.8%). People with missing data on sleep conditions were excluded (*n* = 60), and 2,059 were finally included in this study (Fig. 1).

### Neck pain

Neck pain was assessed using a self-reported questionnaire based on the Comprehensive Survey of Living Conditions [24]. The question was “Have you had symptoms within the last few days? If yes, please check your symptoms (multiple choices were allowed).” There were 28 choices, such as “dizziness”, “irritation”, “head ache”, “low back pain”, and “katakori” (neck pain). The participants who checked “katakori” was considered to have neck pain. We used the data of neck pain at the fourth time point to assess the association between sleep disturbance and neck pain. In addition, we used the data of neck pain at the third and fourth time points to assess the effect of preceding sleep disturbance on the onset of neck pain.

### Sleep disturbance

Sleep disturbance was assessed using the Athens Insomnia Scale (AIS). The AIS is a self-assessment instrument to report any sleep disorders and its validity was shown previously [25]. The AIS consists of eight items rated from 0 to 3 and sleep disturbance was defined as a score of > 6/24 on the AIS [25]. Duration of sleep disturbance at the fourth time point was defined and categorised into four groups as follows: (i) absent, absence of sleep disturbance at the fourth time point; (ii) < 1 year, absence of sleep disturbance at the third time point and presence of sleep disturbance at the fourth time point; (iii) ≥ 1 year and < 2 years, absence of sleep disturbance at the second time point and presence of sleep disturbance at the third and fourth time points; and (iv) ≥ 2 years, presence of sleep disturbance at the second, third, and fourth time points.

The duration of sleep disturbance at the third time point was also defined and categorised into four groups as follows: (1) absent, absence of sleep disturbance at the third time point; (2) < 1 year, absence of sleep disturbance at the second time point and presence of sleep disturbance at the third time point; (3) ≥ 1 year and < 2 years, absence of sleep disturbance at the first time point and presence of sleep disturbance at the second and third time points; (4) and ≥ 2 years, presence of sleep disturbance at the first, second, and third time points.

### Covariates

The following variables at the third or fourth time point were included in the analysis as covariates because they had the potential to be confounding factors: sex, age, body mass index, living area and status, smoking and drinking habits, comorbid conditions, working conditions, walking time per day, economic and psychological conditions, and social network. Psychological condition was assessed using the Kessler Psychological Distress Scale-6 (K6) [26]. K6 consists of six questions for mental illness rated from 0 to 4 and its validity was shown previously [27]. A score of > 10/24 was defined as having psychological distress [28]. Social network was assessed using the Lubben Social Network Scale-6 (LSNS-6) [29]. LSNS-6 consists of six items rated from 0 to 5 and is used as an indicator of social isolation, and its validity was also previously demonstrated [30]. A score of < 12/30 was defined as experiencing social isolation [31]. These variables were categorised as shown in Table 1.

**Table 1:** Baseline characteristics

	Sleep disturbance at the fourth time point			
	n (%)	absence	presence	*P* value
	2,059	1,372	687	
Sex				
Male	911 (44.2)	662 (48.3)	249 (36.2)	< 0.001
Female	1,148 (55.8)	710 (51.7)	438 (63.8)	
Age				
< 65	820 (39.8)	525 (38.3)	295 (42.9)	0.041
≥ 65	1,239 (60.2)	847 (61.7)	392 (57.1)	
Body mass index^a^				
≥ 18.5, < 25	1,240 (60.2)	832 (60.6)	408 (59.4)	0.54
< 18.5	38 (1.8)	22 (1.6)	16 (2.3)	
≥ 25	706 (34.3)	465 (33.9)	241 (35.1)	
Living area				
Ogatsu	857 (41.6)	534 (38.9)	323 (47.0)	< 0.001
Oshika	740 (35.9)	526 (38.3)	214 (31.1)	
Ajishima	137 (6.7)	117 (8.5)	20 (2.9)	
Wakabayashi	325 (15.8)	195 (14.2)	130 (18.9)	
Smoking habits^a^				
Non-smoker	1,656 (80.4)	1,100 (80.2)	556 (80.9)	0.866
Smoker	330 (16.0)	224 (16.3)	106 (15.4)	
Drinking habits^a^				
Non-drinker	1,292 (62.7)	839 (61.2)	453 (65.9)	0.05
< 45.6 g of alcohol/day^b^	438 (21.3)	315 (23.0)	123 (17.9)	
≥ 45.6 g of alcohol/day^b^	161 (7.8)	110 (8.0)	51 (7.4)	
Comorbid conditions				
Hypertension	877 (42.6)	578 (42.1)	299 (43.5)	0.546
Diabetes mellitus	222 (10.8)	151 (11.0)	71 (10.3)	0.643
Myocardial infarction	135 (6.6)	85 (6.2)	50 (7.3)	0.349
Cerebral stroke	31 (1.5)	18 (1.3)	13 (1.9)	0.308
Working status^a^				
Unemployed	1,033 (50.2)	667 (48.6)	366 (53.3)	0.105
Employed	973 (47.3)	671 (48.9)	302 (44.0)	
Walking time/day^a^				
≥ 1 h	594 (28.8)	438 (31.9)	156 22.7)	< 0.001
30 min to < 1 h	752 (36.5)	505 (36.8)	247 (36.0)	
< 30 m	685 (33.3)	408 (29.7)	277 (40.3)	
Living status^a^				
Same house as before the GEJE	670 (32.5)	489 (35.6)	181 (26.3)	0.001
Prefabricated house	83 (4.0)	48 (3.5)	35 (5.1)	
New house	654 (31.8)	419 (30.5)	235 (34.2)	
Others	629 (30.5)	400 (29.2)	229 (33.3)	
Economic condition^a^				
Normal	1,020 (49.5)	798 (58.2)	222 (32.3)	< 0.001
A little hard	488 (23.7)	296 (21.6)	192 (27.9)	
Hard	324 (15.7)	174 (12.7)	150 (21.8)	
Very hard	191 (9.3)	77 (5.6)	114 (16.6)	
Psychological distress^a^				
Absence	1,775 (86.2)	1,289 (94.0)	486 (70.7)	< 0.001
Presence	253 (12.3)	56 (4.1)	197 (28.7)	
Social isolation^a^				
Absence	1,454 (70.6)	1,025 (74.7)	429 (62.4)	< 0.001
Presence	604 (29.3)	347 (25.3)	257 (37.4)	

^a^Because each item has a limited number of respondents, the actual number is not necessarily in accordance with the total

^b^22.8 g of alcohol amount to 1 go or traditional unit of sake (180 ml), which also approximates to two glasses of wine (200 ml), or beer (500 ml) in terms of alcohol content. Categorical values are presented as numbers and percentage (%)

*GEJE* Great East Japan Earthquake

### Statistical analysis

The χ^2^ test was used to compare covariates due to sleep disturbances. Crude and multivariate logistic regression analyses were performed to assess the association between sleep disturbance and neck pain; the results were presented with their respective odds ratios (ORs) and 95% confidence intervals (CIs). In all analyses, the outcome of interest was neck pain at the fourth time point. First, sleep disturbance at the fourth time point was used as the main predictor to assess the association between sleep disturbance and neck pain. Furthermore, the association between the duration of sleep disturbance at the fourth time point and neck pain was assessed. In addition, the participants were divided into subgroups based on age (< 65 years or ≥ 65 years) and sex (male or female), and the association between sleep disturbance and neck pain was also assessed in these subgroups. The Wald test was used to assess the multiplicative interaction between sleep disturbance and age or sex. The covariates were set as variables at the fourth time point in these analyses. Second, participants without neck pain at the third time point were selected, and sleep disturbance at the third time point was used as the main predictor to assess the effect of preceding sleep disturbance on the onset of neck pain. Furthermore, the effect of the duration of sleep disturbance at the third time point on the onset of neck pain was also assessed. The covariates were set as variables at the third time point in these analyses. SPSS (version 24.0: IBM Corp., Armonk. NY) was used for all statistical analyses, and a *p* value of < 0.05, was considered significant.

## Results

The variables divided by sleep disturbances are shown in Table 1. At the fourth time point, 33.4% of the participants had sleep disturbances. The variables associated with sleep disturbance were sex, age, living area, walking time per day, living status, economic condition, psychological distress, and social isolation. The rate of neck pain at the fourth time point was 19.9% (410/2,059). Sleep disturbance was significantly associated with neck pain and adjusted OR (95% CI) in presence of sleep disturbance was 2.45 (1.91–3.15) when the absence of sleep disturbance was the reference. Furthermore, with a longer duration of sleep disturbance, the association between sleep disturbance and neck pain was stronger. Using the absence of sleep disturbance as the reference, adjusted ORs (95% CIs) were 1.84 (1.23–2.75) in “ < 1 year”, 2.41 (1.53–3.81) in “ ≥ 1 year and < 2 years”, and 2.80 (2.09–3.76) in “ ≥ 2 years” (*p* for trend < 0.001) (Table 2). In the stratified analyses, sleep disturbance was also significantly associated with neck pain in each subgroup, and the association was stronger as the duration of sleep disturbance increased. No significant multiplicative interaction was observed between sleep disturbance and age or sex (Tables 3 and 4).

**Table 2:** Association between sleep disturbance and neck pain

	Sleep disturbance at the fourth time point					
	Total	Absence	Presence			*P* value
Participants	2,059	1,372	687			
Neck pain, n (%)	410 (19.9)	192 (14.0)	218 (31.7)			
Crude OR (95% CI)		1 (Ref.)	2.86 (2.29–3.57)			< 0.001
Adjusted OR (95% CI)		1 (Ref.)	2.45 (1.91–3.15)			< 0.001
			duration			
			< 1 year	≥ 1 year, < 2 years	≥ 2 years	*P* for trend
Participants			168	109	410	
Neck pain, n (%)			43 (25.6)	34 (31.2)	141 (34.4)	
Crude OR (95% CI)		1 (Ref.)	2.11 (1.45–3.09)	2.79 (1.81–4.30)	3.22 (2.50–4.16)	< 0.001
Adjusted OR (95%CI)		1 (Ref.)	1.84 (1.23–2.75)	2.41 (1.53–3.81)	2.80 (2.09–3.76)	< 0.001

Adjusted for sex, age, body mass index, living area, smoking habits, drinking habits, comorbid conditions, working status, walking time, living status, subjective economic condition, psychological distress, and social isolation

*OR* Odds ratio

*CI* Confidence interval

**Table 3:** Stratified analysis for age

	Duration of sleep disturbance					
	Total	Absence	< 1 year	1 year, < 2 years	≥ 2 years	*P* for trend
< 65 years old						
Participants	820	525	80	59	156	
Neck pain, n (%)	205 (25.0)	89 (17.0)	25 (31.3)	24 (40.7)	67 (42.9)	
Adjusted OR (95%CI)		1 (Ref.)	2.14 (1.21–3.80)	2.95 (1.58–5.50)	3.16 (2.00–4.99)	< 0.001
≥ 65 years old						
Participants	1,239	847	88	50	254	
Neck pain, n (%)	205 (16.5)	103 (12.2)	18 (20.5)	10 (20.0)	74 (29.1)	
Adjusted OR (95%CI)		1 (Ref.)	1.70 (0.94–3.07)	1.65 (0.77–3.54)	2.64 (1.77–3.93)	< 0.001
					*P*-interaction = 0.256	

Adjusted for sex, body mass index, living area, smoking habits, drinking habits, comorbid conditions, working status, walking time, living status, subjective economic condition, psychological distress, and social isolation

*OR* Odds ratio

*CI* Confidence interval

**Table 4:** Stratified analysis for sex

	Duration of sleep disturbance					
	Total	Absence	< 1 year	1 year, < 2 years	≥ 2 years	*P* for trend
Male						
Participants	911	662	67	39	143	
Neck pain, n (%)	130 (14.3)	64 (9.7)	14 (20.9)	10 (25.6)	42 (29.4)	
Adjusted OR (95%CI)		1 (Ref.)	2.18 (1.11–4.30)	3.01 (1.33–6.78)	3.51 (2.08–5.93)	< 0.001
Female						
Participants	1,148	710	101	70	267	
Neck pain, n (%)	280 (24.4)	128 (18.0)	29 (28.7)	24 (34.3)	99 (37.1)	
Adjusted OR (95%CI)		1 (Ref.)	1.63 (0.98–2.71)	2.23 (1.26–3.95)	2.59 (1.80–3.72)	< 0.001
					*P*-interaction = 0.347	

Adjusted for age, body mass index, living area, smoking habits, drinking habits, comorbid conditions, working status, walking time, living status, subjective economic condition, psychological distress, and social isolation

*OR* Odds ratio

*CI* Confidence interval

In participants without neck pain at the third time point, the rate of onset of neck pain at the fourth time point was 10.6% (174/1,647). Sleep disturbance at the third time point was significantly associated with the onset of neck pain at the fourth time point and adjusted OR (95% CI) in presence of sleep disturbance was 2.47 (1.72–3.55) when the absence of sleep disturbance was the reference. Moreover, with a longer duration of sleep disturbance, the association between sleep disturbance at the third time point and the onset of neck pain was stronger. Using the absence of sleep disturbance as the reference, adjusted ORs (95% CIs) were 1.86 (1.08–3.20) in “ < 1 year”, 2.39 (1.22–4.70) in “ ≥ 1 year and < 2 years”, and 3.00 (1.94–4.65) in “ ≥ 2 years” (*p* for trend < 0.001) (Table 5).

**Table 5:** Association between preceding sleep disturbance and onset of neck pain

	Sleep disturbance at the third time point					
	Total	Absence	Presence			*P* value
Participants without neck pain at the third time point	1,647	1,178	469			
Onset of neck pain at the fourth time point, n (%)	174 (10.6)	88 (7.5)	86 (18.3)			
Crude OR (95% CI)		1 (Ref.)	2.78 (2.02–3.83)			< 0.001
Adjusted OR (95%CI)		1 (Ref.)	2.47 (1.72–3.55)			< 0.001
				duration		
			< 1 year	≥ 1 year, < 2 years	≥ 2 years	*P* for trend
Participants without neck pain at the third time point			142	75	252	
Onset of neck pain at the fourth time point, n (%)			21 (14.8)	13 (17.3)	52 (20.6)	
Crude OR (95% CI)		1 (Ref.)	2.15 (1.29–3.59)	2.60 (1.38–4.91)	3.22 (2.21–4.68)	< 0.001
Adjusted OR (95%CI)		1 (Ref.)	1.86 (1.08–3.20)	2.39 (1.22–4.70)	3.00 (1.94–4.65)	< 0.001

Adjusted for sex, age, body mass index, living area, smoking habits, drinking habits, comorbid conditions, working status, walking time, living status, subjective economic condition, psychological distress, and social isolation

*OR* Odds ratio

*CI* Confidence interval

## Discussion

The present study revealed that sleep disturbance was significantly associated with neck pain, which was stronger with a longer duration of sleep disturbance. Furthermore, preceding sleep disturbance was significantly associated with the onset of neck pain, and the association was stronger as the duration of sleep disturbance increased.

Nowadays, an increasing number of reports have shown an association between sleep disturbance and pain, which is observed in several types of pain, such as fibromyalgia, rheumatoid arthritis, orofacial pain, and low back pain [10, 11, 32, 33]. Regarding neck pain, although the number of reports is few, some authors have also shown an association with sleep disturbance. Artner et al. reported that approximately 41% of people with neck pain presented with sleep disturbance [13].　Kovacs et al. showed that improvement of neck pain was poorer in patients with sleep disturbance than in those without sleep disturbance [15]. The present study reported that people with sleep disturbance had a significantly higher rate of neck pain than those without sleep disturbance, which also indicated the significant association between sleep disturbance and neck pain. In addition, Valenza et al. showed that the intensity of neck pain was higher with worse sleep quality, which implied that the association between sleep disturbance and neck pain was dose-dependent. We hypothesised that the duration of sleep disturbance was associated with neck pain, which has not been reported to date. The results of this study clearly showed that the rate of neck pain was higher with a longer duration of sleep disturbance. Although the effect of the duration of sleep disturbance on pain has been rarely investigated, some studies have shown that the association is stronger with the increased duration or frequency of sleep disturbance among people with fibromyalgia or low back pain [34–36]. Sleep disturbance is associated with neck pain, and long-lasting sleep disturbance is considered to strengthen the association. Further, the stratified analyses also showed that the association between sleep disturbance and neck pain was significant, and the association was stronger as the duration of sleep disturbance was longer in each group, which indicated the robustness of the results in this study.

Regarding the association between sleep disturbance and pain, their bidirectional effect has been reported [32]. A previous study reported that preceding musculoskeletal pain including neck pain was associated with the onset of sleep disturbance [19]. Conversely, some longitudinal studies have reported that preceding sleep disturbance is associated with the onset of musculoskeletal pain [34, 37], which has also been reported after natural disasters [38–40]. Regarding neck pain, a few reports have shown the effect of sleep disturbance on neck pain in longitudinal studies [16, 18]. Rasmussen et al. showed that sleep disturbance was associated with neck pain four years later among the working-age population, and the association was stronger along with worse sleep disturbance [16]. Mork et al. also reported that sleep disturbance was associated with neck pain 10 years later among the general population and the rate of neck pain was higher among people with more frequent sleep disturbance [18]. The present study assessed the people without neck pain at baseline, which could indicate that preceding sleep disturbance was significantly associated with the onset of neck pain 1 year later among people after a natural disaster. In addition, the association was stronger as the duration of sleep disturbance increased. Experimental human and animal studies indicated that sleep disturbance changed the descending pain inhibitory control system and prevented the analgesic action of endogenous opioids, which resulted in a reduction in the pain perception threshold [10, 32, 41]. This effect is considered to be stronger with worse or longer sleep disturbance. The present study showed that sleep disturbance affected the onset of neck pain in a dose-dependent manner. Clinicians should consider this association to effectively treat patients with neck pain.

The present study had some limitations. First, we did not have data on people who did not participate in this study. Second, the previous history and intensity of neck pain, and self-reported disability due to neck pain were not assessed. These information are important to define neck pain and its severity more accurately and should be investigated to assess the association between sleep disturbance and neck pain in future studies. Finally, the participants of the present study were people living in disaster-stricken areas after the GEJE; thus, the generalisability of the results of this study may not be fully applicable.

In conclusion, sleep disturbance was associated with neck pain among people living in disaster-affected areas after the GEJE, and the association was stronger as the duration of sleep disturbance increased. Furthermore, preceding sleep disturbance was associated with the onset of neck pain, and the effect was stronger with longer durations of sleep disturbance.

## Data Availability

The datasets used and/or analysed during the current study are available from the corresponding author on reasonable request.

## Abbreviations

GEJE: Great East Japan Earthquake
OR: Odds ratio
95% CI: 95% Confidence interval
AIS: Athens Insomnia Scale
K6: Kessler Psychological Distress Scale-6
LSNS-6: Lubben Social Network Scale-6
